# Stability of Principal Hydrolysable Tannins from *Trapa taiwanensis* Hulls

**DOI:** 10.3390/molecules24020365

**Published:** 2019-01-21

**Authors:** Ching-Chiung Wang, Hsyeh-Fang Chen, Jin-Yi Wu, Lih-Geeng Chen

**Affiliations:** 1 School of Pharmacy, College of Pharmacy, Taipei Medical University, Taipei 11031, Taiwan; crystal@tmu.edu.tw; 2 Traditional Herbal Medicine Research Center, Taipei Medical University Hospital, Taipei 11031, Taiwan; 3 Department of Microbiology, Immunology and Biopharmaceuticals, College of Life Sciences, National Chiayi University, Chiayi 60004, Taiwan; snowfang@ccpc.com.tw (H.-F.C.); jywu@mail.ncyu.edu.tw (J.-Y.W.)

**Keywords:** *Trapa taiwanensis* Nakai, hydrolysable tannin, stability, gallotannin, ellagitannin

## Abstract

The fruit and hulls of the water caltrop (*Trapa taiwanensis* Nakai) are used as hepatoprotective herbal tea ingredients in Taiwan. The stability of hydrolysable tannins in herbal drinks has rarely been reported. In the present study, two hydrolysable tannins, tellimagrandin II (TGII) and 1,2,3,4,6-pentagalloylglucopyranose (PGG), were isolated from water caltrop hulls. The stability of the two compounds was evaluated by treatment with various pH buffer solutions, simulated gastric fluid and intestinal fluid, different temperatures, and photo-irradiation at 352 nm in different solvents. Results showed that TGII and PGG were more stable in a pH 2.0 buffer solution (with 91.88% remaining) and in a water solution with 352 nm irradiation (with 95% remaining). TGII and PGG were more stable in methanol or ethanol solutions (with >93.69% remaining) than in an aqueous solution (with <43.52% remaining) at 100 °C. In simulated gastric fluid, more than 96% of the hydrolysable tannins remained after incubation at 37 °C for 4 h. However, these hydrolysable tannins were unstable in simulated intestinal fluid, as after incubation at 37 °C for 9 h, the content of TGII had decreased to 31.40% and of PGG to 12.46%. The synthetic antioxidants, butyl hydroxy anisole (BHA), di-butyl hydroxy toluene (BHT), and propyl gallate, did not exhibit photoprotective effects on these hydrolysable tannins. However, catechin, a natural antioxidant, displayed a weak photoprotective effect. Ascorbic acid had a short-term thermal-protective effect but not a long-term protective effect. The different stability properties of hydrolysable tannins in solutions can be used in the development of related herbal teas in the future.

## 1. Introduction

Tannins belong to water-soluble polyphenols and are present in many vegetables and herbal teas [1]. They can be classified into hydrolysable, condensed, and complex tannins [1]. Recently, herbal teas with several health benefits, such as antioxidative, hepatoprotective, and hypolipidemic activities, have become popular in Taiwan [2]. Catechins, hydrolysable tannins, flavonoids, anthocyanins, and chlorogenic acid derivatives are the major active ingredients in herbal teas. For convenience, bottled herbal tea drinks are popular and are being produced worldwide. The contents of bioactive polyphenols influence the shelf-life of drinks. However, the stability of these polyphenols in aqueous solution has rarely been reported [3,4].

Water caltrop (*Trapa taiwanensis* Nakai, Trapaceae), a floating plant that grows in shallow water, is widely used in folk medicine for treating diarrhea and dysentery. Hot water decoctions of water caltrop fruit or hulls are used as herbal drinks with hepatoprotective effect in Taiwan [5]. Tea bags containing water caltrop hulls are also commercially available in Taiwan. Water caltrop was reported to have hepatoprotective, antioxidative, antibacterial, anti-inflammatory, hypoglycemic, and other healthy functions [5,6,7,8,9,10,11,12,13,14,15,16,17,18,19,20,21,22]. Hydrolysable tannins are regarded as the active compounds in water caltrop hulls [5,8,11,21,22]. Hulls of *T. taiwanensis* have the potential to be developed as herbal tea drink ingredients.

In the present study, we isolated two major hydrolysable tannins components, tellimagrandin II (TGII) and 1,2,3,4,6-pentagalloylglucopyranose (PGG) from water caltrop hulls, which are respectively classified as ellagitannin and gallotannin. These two hydrolysable tannins were subjected to different pH solutions, simulated gastric and intestinal fluids, different temperatures, and photo-irradiation, and the protective effects of antioxidants were evaluated to determine the stabilities of these two hydrolysable tannins in aqueous solutions.

## 2. Results

### 2.1. Isolation of Hydrolysable Tannins from T. taiwanensis Hulls

**Figure 1:**
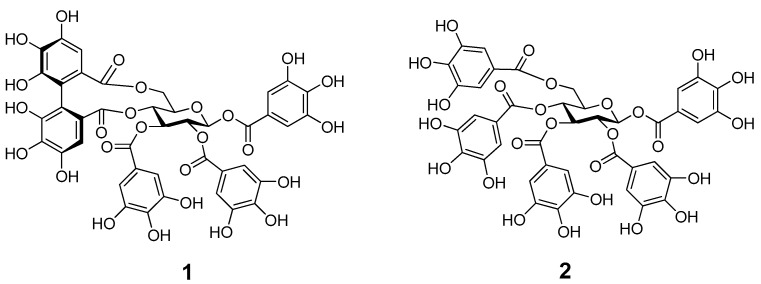
Two hydrolysable tannins, tellimagrandin II (TGII, **1**) and 1,2,3,4,6-pentagalloylglucopyranose (PGG, **2**), isolated from *Trapa taiwanensis* hulls.

Two major hydrolysable tannins, TGII and PGG, were isolated from the ethyl acetate (EtOAc) fraction of T. taiwanensis hulls, and their structures are shown in Figure 1 (Figure S1). Both compounds have a galloyl ester linkage with glucose. We used the two compounds to estimate their stability in different pH solutions, in simulated gastric fluid and intestinal fluid, and when subjected to various light and thermal conditions.

### 2.2. The Stability of Hydrlysable Tannins in Different pH Solutions

**Figure 2:**
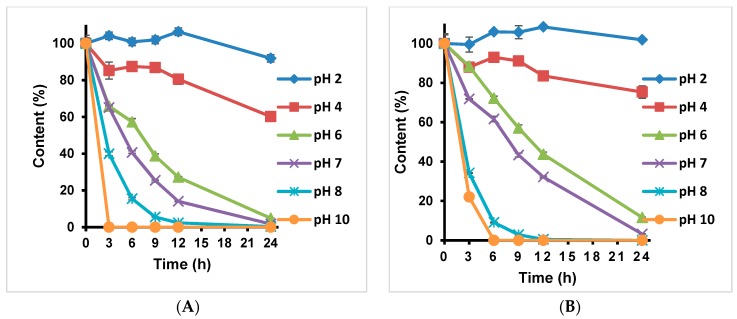
The pH stability test of hydrolysable tannins of TGII (**A**) and PGG (**B**), respectively. These hydrolysable tannins were treated with various pH values. *n* = 3; values are presented as the mean ± standard deviation.

TGII and PGG were dissolved in pH 0.1 M buffer solutions to give a final concentration of 1.0 mg/mL and incubated in a dry bath for 24 h. Results are shown in Figure 2 (Table S1). These hydrolysable tannins were more stable in strongly acidic conditions (pH 2.0 and 4.0) than in weakly acidic, neutral, and basic conditions (pH 6.0, 7.0, 8.0, and 10.0). TGII was more unstable than PGG. After 3 h of incubation in a pH 10.0 buffer solution, TGII had totally degraded, but 20% of PGG remained. TGII and PGG were more stable in a pH 2.0 buffer solution (with 91.88% remaining).

### 2.3. The Stability of Hydrlysable Tannins in Simulated Gastric Fluid and Intestinal Fluid

**Figure 3:**
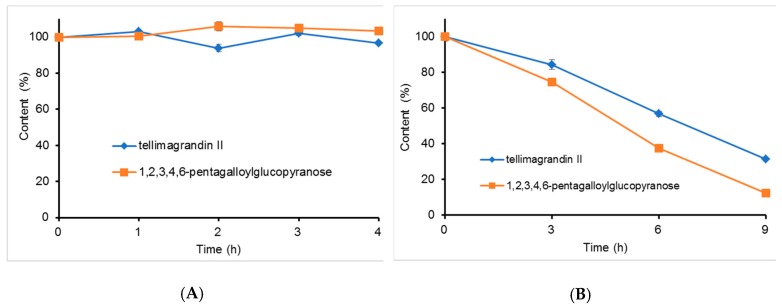
Simulated gastric fluid (**A**) and simulated intestinal fluid (**B**) stability tests of the hydrolysable tannins of TGII and PGG. *n* = 3; values are presented as the mean ± standard deviation.

TGII and PGG were dissolved in simulated gastric fluid and intestinal fluid to a final concentration of 1.0 mg/mL and were then incubated in a dry bath for 4 and 9 h. These incubation times respectively mimicked the physiological condition of food retention times in the stomach and intestines. Results are shown in Figure 3. TGII and PGG were more stable in simulated gastric fluid (pH 1.2) than simulated intestinal fluid (pH 7.5). After 4 h of incubation in simulated gastric fluid, more than 96% of TGII and PGG remained (Figure 3A, Table S2). However, in simulated intestinal fluid TGII and PGG quickly degraded. After 9 h of incubation in simulated intestinal fluid, only 31.40% and 12.46% of TGII and PGG remained, respectively (Figure 3B, Table S2).

### 2.4. The Photostability of Hydrlysable Tannins

**Figure 4:**
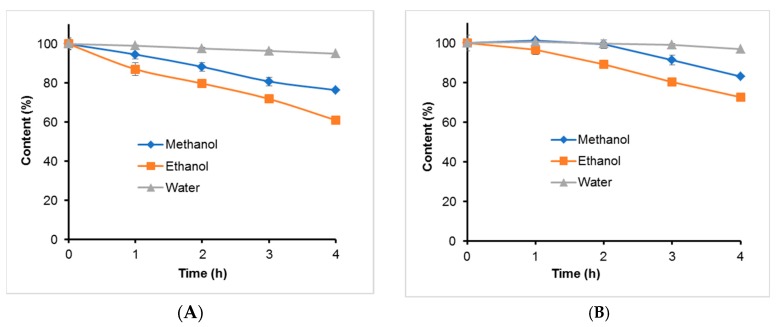
Photostability test of hydrolysable tannins of (**A**) TGII and (**B**) PGG. The sample solution was irradiated with an ultraviolent lamp of a photochemical reactor (8 W × 16 = 128 W) at 352 nm and a distance of about 3.2 cm for 4 h. *n* = 3; values are presented as the mean ± standard deviation.

TGII and PGG were dissolved in different solvents (1.0 mg/mL) and placed in a photochemical reactor for irradiation with an ultraviolent (UV) lamp at 352 nm (8 W × 16 = 128 W) for 4 h. Results are shown in Figure 4 (Table S3). These hydrolysable tannins were more stable in water (with 95% remaining) than methanol or ethanol. PGG was more stable than TGII. These hydrolysable tannins dissolved in ethanol (EtOH) were more sensitive to UV irradiation. After 4 h, 60.98% of TGII and 72.74% of PGG remained. Four antioxidants (butyl hydroxy anisole (BHA), di-butyl hydroxy toluene (BHT), propyl gallate, and catechin) were individually added to the sample solution to test their light protective effects. Only propyl gallate and catechin showed weak irradiation protective effects for PGG (Table 1).

### 2.5. The Thermal Stability of Hydrlysable Tannins

**Figure 5:**
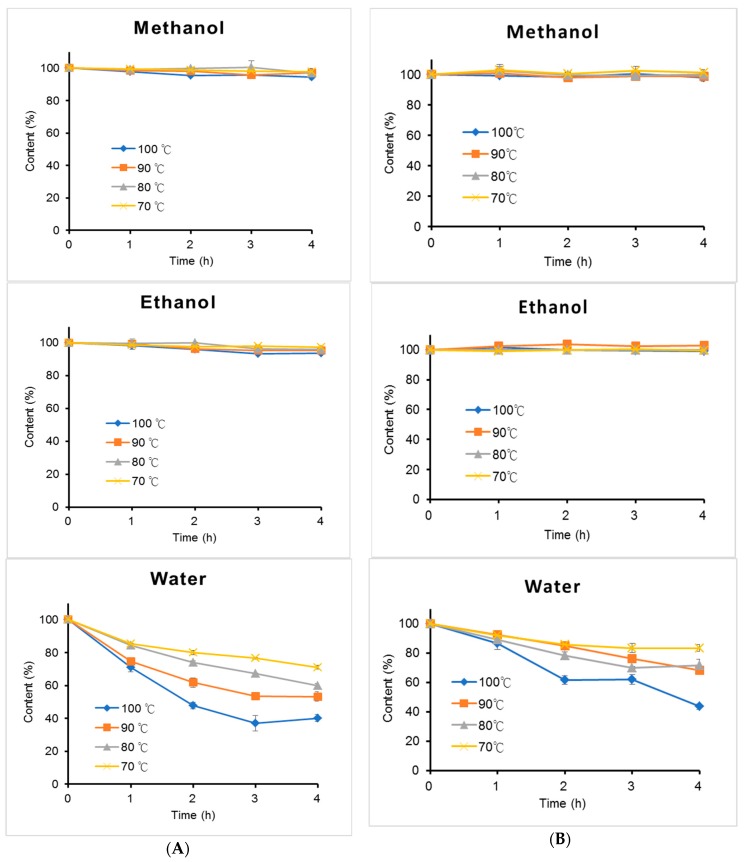
Thermal stability test of hydrolysable tannins of (**A**) TGII and (**B**) PGG in methanol, ethanol, and water solutions. Sample solutions were placed in a dry bath at 70, 80, 90, and 100 °C for 4 h. *n* = 3; values are presented as the mean ± standard deviation.

TGII and PGG dissolved in methanol, ethanol, or water (1.0 mg/mL) were incubated at different temperatures in a dry bath for 4 h. Results are shown in Figure 5 (Table S4). These hydrolysable tannins were more stable in the methanol or ethanol solution than in the aqueous solution at temperatures from 70 to 100 °C. When the temperature increased in the aqueous solution, the hydrolysable tannins were more unstable. PGG was more stable than TGII in the aqueous solution. TGII and PGG were more stable in the methanol and ethanol solutions (with >93.69% remaining) than in the aqueous solution (with <43.52% remaining) at 100 °C.

### 2.6. Protective Effect of Ascorbic Acid on Thermal Stability

**Figure 6:**
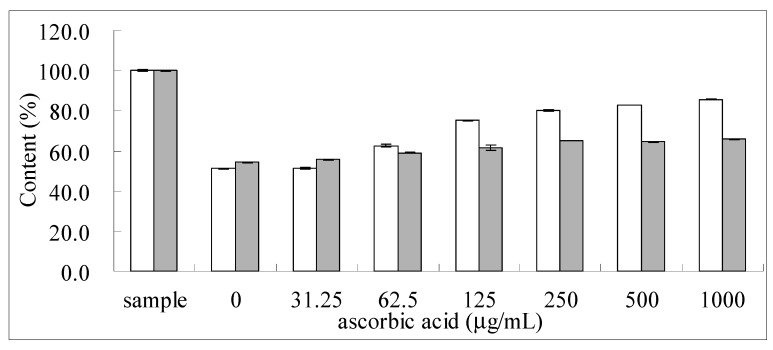
Protective effects of different concentrations of ascorbic acid on TGII (□) and PGG (■) in a dry bath at 100 °C for 4 h. *n* = 3; values are presented as the mean ± standard deviation.

Different concentrations of ascorbic acid (0~1000 μg/mL) were added to the sample solution to evaluate the protective effect on hydrolysable tannins in 100 °C aqueous solutions. Ascorbic acid protected TGII and PGG from thermal degradation (Figure 6, Table S5). When a high concentration of ascorbic acid (1 mg/mL) was added to a TGII solution and incubated at 100 °C for 4 h, the content increased from 51.28% (without ascorbic acid) to 85.55%. However, when a high concentration of ascorbic acid (1 mg/mL) was added to a PGG solution and incubated at 100 °C for 4 h, the content moderately increased from 54.29% (without ascorbic acid) to 65.95%. Long-term storage with ascorbic acid was evaluated at different temperatures (4 °C and 25 °C) for 4 weeks. Results are shown in Table 2. When aqueous solutions of hydrolysable tannins were stored at 4 °C for 4 weeks, the contents of TGII and PGG moderately decreased. However, when stored at 25 °C, contents of TGII and PGG significantly decreased.

## 3. Discussion

The structures of hydrolysable tannins contain several ester linkages which are easily cleaved when in a basic solution or at higher temperatures. In the present study, we found two types of hydrolysable tannins that were more resistant in an acidic solution than a basic solution. Gallotannins are more stable than ellagitannins in acidic or basic solutions. Tuominen and Sundman reported that hydrolysable tannins were unstable in a basic condition, and the degradation products were formed by hydrolysis, deprotonation, and oxidation [4]. Ellagic acid can also be formed by gallotannins in a basic condition [4]. Ellagitannin metabolites, urolithins, with potent antioxidative activities, were found in plasma after the oral administration of geraniin and other ellagitannin-rich plant leaves to animals [1]. Urolithin B was more stable than urolithin A and ellagic acid in simulated gastrointestinal fluid and fecal fermentation [3]. Ellagitannins are hydrolyzed to produce ellagic acid in the small intestine, and then ellagic acid is metabolized to urolithins by intestinal bacterial fermentation.

Beverages and bottled herbal tea drinks are becoming more popular worldwide. Such drinks are stored in transparent plastic bottles and sold in convenience stores. People do not need to waste time preparing herbal decoctions themselves. However, herbal teas in a liquid state are more unstable than in tea bags or as a powder. UV radiation can be classified into UVA (320~400 nm), UVB (280~320 nm), and UVC (100~280 nm) according to different wavelengths [23]. UVA can be divided into UVA-1 (340~400 nm) and UVA-2 (320~340 nm). The ability of UVA-1 to penetrate is stronger than that of UVB or UVC. In the present study, a 352-nm UV lamp belonging to UVA-1 region was used to study the photoprotective effect of antioxidants [23]. UV can generate radicals which degrade hydrolysable tannins. The synthetic antioxidants, BHA, BHT, and propyl gallate, have free radical-scavenging effects which can protect compounds from oxidation and are widely used in processed food products. However, as shown in Table 1, BHA, BHT, and propyl gallate did not exert photoprotective effects on hydrolysable tannins. But catechin, a natural antioxidant, displayed a weak photoprotective for PGG but not TGII. Therefore, UV irradiation may cause cleavage of chemical bonds of hydrolysable tannins which cannot be prevented by antioxidants.

Ascorbic acid was used in the aqueous solution as an antioxidant. In Figure 6, the short-term thermal protective effect of ascorbic acid was significant for both hydrolysable tannins. However, in Figure 2, the long-term protective effect of ascorbic acid was not significant. The ascorbic acid concentrations that remained in the two temperature conditions (data not shown) significantly differed. Ascorbic acid in the 4 °C solution was higher than that in the 25 °C solution. After 4 weeks of storage at 4 and 25 °C, ascorbic acid concentrations in the two solutions were 866~815 and 28~24 μg/mL, respectively. It was interesting that ascorbic acid was exhausted at the high temperature and enhanced degradation of the hydrolysable tannins (Table 2). The reason may have been oxidative stress produced by the oxidized ascorbic acid. These results indicated that adding ascorbic acid to hydrolysable tannin-rich herbal teas to prevent oxidation is not effective. Adjusting the pH to be mildly acidic, protecting hydrolysable tannin-rich herbal teas from light, and storing them at 4 °C can improve their shelf life. The different stability properties of TGII and PGG can be used for developing related herbal drinks in the future.

## 4. Materials and Methods

### 4.1. General

^1^H (500 MHz) and ^13^C nuclear magnetic resonance (NMR) (125 MHz) spectra were measured on a Bruker Avance DRX 500 instrument (Bruker, Billerica, MA, USA). The chemical shifts were calibrated by the actone-d_6_ solvent signal and are given in δ (ppm) values. Acetonitrile, l-ascorbic acid, sodium carbonate, sodium chloride, and sodium hydroxide were purchased from J.T. Baker (Phillipsburg, NJ, USA). (+)-Catechin, butyl hydroxy anisole (BHA), di-butyl hydroxy toluene (BHT), boric acid, hydrochloric acid, potassium chloride, potassium biphthalate, pancreatin from porcine pancreas, and pepsin were purchased from Sigma-Aldrich (St. Louis, MO, USA). *n*-Propyl 3,4,5-trihydroxybenzoate (propyl gallate) and trifluoroacetic acid (TFA) were purchased from Alfa Aesar (Tewksbury, MA, USA). Ultrapurified water (>18 mΩ·cm) was produced by EASYpure LF (Barnstead, Dubuque, IA, USA). Column chromatography was carried out on Diaion HP-20 gel (Mitsubishi Chemical Industry, Tokyo, Japan) and LiChroprep RP-18 gel (40~63 µm, Merck, Darmstadt, Germany).

### 4.2. Plant Material

The fruit of *T. taiwanensis* was purchased in December 2006 at Guantian, Tainan. Hulls of *T. taiwanensis* were separated and air blow-dried below 40 °C. A voucher specimen was deposited in the Department of Microbiology, Immunology and Biopharmaceuticals, College of Life Sciences, National Chiayi University (Chiayi, Taiwan).

### 4.3. Isolation of Hydrolysable Tannins from T. taiwanensis Hulls

Dried hulls of *T. taiwanensis* (2.0 kg) were pulverized and macerated with methanol (20 L × 5) at room temperature. After being concentrated, the methanol extract (318 g) was dissolved in water and extracted with EtOAc. The EtOAc layer (45 g) was used to separate TGII and PGG. The EtOAc layer was chromatographed on a Diaion HP-20 column (9 cm i.d. × 40 cm) sequentially eluted with H_2_O (5 L), 20% MeOH (5 L), 40% MeOH (10 L), 60% MeOH (5 L), and 100% MeOH (5 L). A portion (9.2 g) of the Diaion HP-20 column 40% MeOH eluate was purified on a LiChroprep RP-18 column (2.5 cm i.d. × 50 cm) eluted with 0.05% TFA-CH_3_CN (85:15) to obtain TGII (4.95 g, yield 0.25%) and PGG (2.23 g, yield 0.11%).

TGII. ^1^H-NMR (500 MHz, acetone-*d*_6_) δ: 7.12 (2H, s, galloyl H), 7.01 (2H, s, galloyl H), 6.97 (2H, s, galloyl H), 6.66 (1H, s), 6.48 (1H, s), 6.20 (1H, d, *J* = 8.4 Hz, Glc H-1), 5.84 (1H, t, *J* = 9.6Hz, Glc H-3), 5.60 (1H, dd, *J* = 8.4, 9.6 Hz, Glc H-2), 5.36 (1H, dd, *J* = 6.6, 13.4 Hz, Glc H-6), 5.22 (1H, t, *J* = 9.6 Hz, H-4), 4.55 (1H, dd, *J* = 6.2, 9.6 Hz, Glc H-5), 3.89 (1H, d, 13.4 Hz, Glc H-6). ^13^C-NMR (125 MHz, acetone-d_6_) δ: 167.3, 166.9, 165.5, 164.9, 164.2 (C=O), 145.3, 145.1, 144.9, 144.4, 144.3, 143.6, 138.9, 138.5, 138.3, 135.7, 135.5, 125.6, 125.0, 119.6, 119.5, 118.9, 114.9, 114.8, 109.4, 0.9.3, 109.2, 107.3, 107.0, 92.8 (Glc C-1), 72.4 (Glc C-3), 72.2 (Glc C-5), 71.0 (Glc C-2), 69.9 (Glc C-4), 62.2 (Glc C-6).

PGG. ^1^H-NMR (500 MHz, acetone-*d*_6_) δ: 7.17, 7.11, 7.05, 7.01, 6.97 (each 2H, galloyl-H), 6.33 (1H, d, *J* = 8.3 Hz, Glc H-1), 6.01 (1H, t, *J* = 9.7 Hz, Glc H-3), 5.65 (1H, t, *J* = 9.7 Hz, Glc H-4), 5.62 (1H, dd, *J* = 8.3, 9.7 Hz, Glc H-2), 4.56 (1H, m, Glc H-5), 4.53 (1H, brd, *J* = 12.0 Hz, Glc H-6), 4.40 (1H, dd, *J* = 5.0, 12.0 Hz, Glc H-6). ^13^C-NMR (125 MHz, acetone -d_6_) δ: 166.5, 166.0, 165.8, 165.7, 165.1 (C=O), 146.3, 146.1, 146.1, 146.1, 146.0 (galloyl C-3, 5), 139.9, 139.4, 139.4, 139.2, 139.1 (galloyl C-4), 121.5, 120.8, 120.7, 120.0 (galloyl C-1), 110.5, 110.4, 110.3, 110.2 (galloyl C-2, 6), 93.4 (Glc C-1), 74.0 (Glc C-3), 73.4 (Glc C-5), 71.8 (Glc C-2), 69.4 (Glc C-4), 62.9 (Glc C-6).

### 4.4. Stability Studies

#### 4.4.1. Preparation of Solutions

TGII and PGG were dissolved in purified water to a final concentration of 2 mg/mL as stock solutions. The 0.2 M buffered solutions at pH 2, 4, 6, 7, 8, and 10 were prepared according USP38 [24].

#### 4.4.2. Stability of pH

TGII and PGG stock solutions were dissolved in an equal volume of different pH aqueous buffer solutions to a final concentration of 1 mg/mL. The solution was dispensed in a 2-mL glass vial and kept in a 37 °C dry bath for 24 h. Vials were taken every 3 h and analyzed by high-performance liquid chromatography (HPLC) to calculate the content.

#### 4.4.3. Simulated Gastric Fluid Stability

Simulated gastric fluid (pH 1.2) was prepared by dissolving 2.0 g NaCl, 3.2 g pepsin, and 7.0 mL of 37% HCl in 1 L of purified water [24]. TGII and PGG stock solutions were dissolved in an equal volume of simulated gastric fluid or simulated intestinal fluid to a final concentration of 1 mg/mL. The solution (1 mL) was dispensed in a 2-mL glass vial and kept in a 37 °C dry bath for 4 h. Vials were taken every 1 h, and 0.5 mL 0.2 N Na_2_CO_3_ was added to neutralize the solution. The neutralized solution was passed through a Sep-Pak^®^ Plus C18 cartridge (55~105 µm, Waters, Milford, MA, USA) washed with 10 mL water and then eluted with 10 mL MeOH. The MeOH eluate was analyzed by HPLC to calculate the content.

#### 4.4.4. Simulated Intestinal Fluid Stability

Simulated intestinal fluid was prepared by dissolved 6.8 g KH_2_PO_4_, 190 mL of a 0.2 N NaOH solution, and 10 g pancreatin in 650 mL of purified water. The pH was adjusted with 0.2 N NaOH to 7.5 ± 0.1, and water was added to a final volume of 1 L [24]. TGII and PGG stock solutions were dissolved in an equal volume of simulated intestinal fluid to a final concentration of 1 mg/mL. The solution (1 mL) was dispensed in a 2-mL glass vial and kept in a 37 °C dry bath for 9 h. Vials were taken every 3 h, and 0.5 mL of water was added. The solution was passed through a Sep-Pak Plus C18 cartridge (55~105 µm, Waters) washed with 10 mL water and then eluted with 10 mL MeOH. The MeOH eluate was analyzed by HPLC to calculate the content.

#### 4.4.5. Photostability

The hydrolysable tannins (1 mg/mL) were dissolved in different solvents (methanol, ethanol, and water) in a quartz glass test tube (1 cm i.d.) and then treated with a 352-nm UV lamp of a photochemical reactor (8 W × 16 = 128 W) at a distance of about 3.2 cm for 4 h. An aliquot of the solution was taken every 1 h and analyzed by HPLC to calculate the content. Four antioxidants (BHA, BHT, propyl gallate, and catechin) were added to the sample solution (1 mg/mL EtOH) to test the light protective effect.

#### 4.4.6. Temperature Stability

The hydrolysable tannins (1 mg/mL) were dissolved in different solvents (methanol, ethanol, and water) and placed in a dry bath at different temperatures (70, 80, 90, and 100 °C) for 4 h. All data were from triplicate confirmation tests. An aliquot of the solution was taken and analyzed by HPLC to calculate the content.

#### 4.4.7. Protective Effect of Ascorbic Acid Against Thermal Degradation

Ascorbic acid (0~1000 μg/mL) was added to the sample solution to evaluate the protective effect on aqueous solutions of the hydrolysable tannins at 100 °C. Long-term storage with ascorbic acid was evaluated at different temperatures (4 and 25 °C) for 4 weeks.

### 4.5. HPLC analysis of TGII and PGG

HPLC equipment was composed of a Waters 1525 binary HPLC pump, an in-line degasser AF, a 2487 dual l absorbance detector, a 717 plus autosampler, and Millennium 32 vs. 3.20 software (Milford, MA, USA). The LiChrospher RP-18e column (4.0 mm i.d. × 250 mm, 5 μm, Merck) was used for the HPLC analysis. The mobile phase consisted of water with 0.05% TFA-acetonitrile (85:15). The flow rate was 1.0 mL/min, and 10 μL was injected into the column. The column temperature was maintained at 40 °C. Chromatograms were detected by absorbance at 280 nm. Three replicates were performed. The retention times for TGII and PGG were 9.91 and 11.47 min, respectively. The peak area of the compound at 0 h of treatment was regarded as a content of 100%.

### 4.6. Statistical Evaluation

Data are presented as mean ± standard deviation. Student’s *t*-test was used to compare different contents between treated and untreated samples.

## 5. Conclusions

In conclusion, we investigated the stability of TGII and PGG in solutions of various conditions. Both hydrolysable tannins were quite stable in a pH 2.0 buffer solution and unstable in neutral or basic buffer solutions. In simulated gastric (pH 1.2) and intestinal fluids (pH 7.5), the two hydrolysable tannins also presented similar pH stability properties. The photostability test displayed that the two hydrolysable tannins dissolved in water were stable under UV 352 nm irradiation for 4 h but unstable in methanol and ethanol solutions. The photodegradation of PGG was slightly prevented by propyl gallate and catechin. As to the thermal stability of the two hydrolysable tannins, the contents decreased as the storage temperature increased. Their thermal degradation at 100 °C for 4 h was prevented by ascorbic acid. At room temperature or at refrigerated conditions, adding ascorbic acid did not inhibit the degradation effect. We hope the above results are helpful for the future production of herbal tea drinks.

## Figures and Tables

**Table 1:** Protective effects of various antioxidants against irradiation with UV light at 352 nm.

Irradiation time (h)	Content (%)	
	0	4
TGII	100.00 ± 0.08	66.02 ± 1.60
TGII + BHA	100.00 ± 0.11	63.27 ± 0.37
TGII + BHT	100.00 ± 0.19	64.64 ± 0.15
TGII + propyl gallate	100.00 ± 1.16	51.62 ± 0.54 **
TGII + catechin	100.00 ± 0.44	57.12 ± 0.30 **
PGG	100.00 ± 3.15	70.79 ± 2.92
PGG + BHA	100.00 ± 0.41	66.40 ± 0.91
PGG + BHT	100.00 ± 4.27	52.80 ± 1.03
PGG + propyl gallate	100.00 ± 0.08	78.91 ± 2.20 **
PGG + catechin	100.00 ± 0.28	77.43 ± 2.17 **

These hydrolysable tannins were dissolved in ethanol EtOH (1.0 mg/mL), and the final concentration of each antioxidant was 1.0 mg/mL. *n* = 3; values are represented as the mean ± standard deviation. BHA: butyl hydroxy anisole; BHT: di-butyl hydroxy toluene. ** *p* < 0.01 compared to the untreated sample.

**Table 2:** Protective effects of ascorbic acid on TGII and PGG during storage at 4 and 25 °C.

**Ascorbic Acid (mg/mL)**	**TGII Content (%) ^1^**			
	4 °C		25 °C	
	0	1.0	0	1.0
Time (week)				
0	100.00 ± 1.02	100.00 ± 0.26	100.00 ± 1.02	100.26 ± 0.26
1	101.15 ± 0.35	98.31 ± 0.19	85.24 ± 0.69	83.75 ± 0.73
2	100.05 ± 0.80	95.78 ± 0.50	77.37 ± 1.57	75.56 ± 0.66
3	97.07 ± 0.71	92.88 ± 0.13	71.86 ± 0.84	68.18 ± 0.91
4	96.41 ± 1.51	90.92 ± 0.47	74.29 ± 0.60	52.54 ± 0.49
**Ascorbic Acid (mg/mL)**	**PGG Content (%) ^1^**			
	4 °C		25 °C	
	0	1.0	0	1.0
Time (week)				
0	100.00 ± 0.66	100.00 ± 0.25	100.00 ± 0.66	100.00 ± 0.25
1	100.30 ± 0.50	97.83 ± 0.06	95.89 ± 1.42	73.76 ± 0.31
2	100.13 ± 0.57	92.93 ± 0.81	93.47 ± 0.85	73.23 ± 0.47
3	100.35 ± 0.74	91.89 ± 0.74	86.82 ± 0.69	44.57 ± 0.11
4	104.18 ± 1.37	97.50 ± 0.43	91.82 ± 15.1	58.05 ± 0.47

^1^ These hydrolysable tannins were dissolved in an aqueous solution (1.0 mg/mL). *n* = 3; values are presented as the mean ± standard deviation.

## Supplementary Materials

The following are available online, Figure S1: The HPLC chromatograms of TGII (A) and PGG (B). Table S1: The pH stability test of hydrolysable tannins of TGII and PGG. These hydrolysable tannins were treated with various pH values. Table S2: Simulated gastric fluid and simulated intestinal fluid stability tests of the hydrolysable tannins of TGII and PGG. Table S3: Photostability test of hydrolysable tannins of TGII and PGG. Table S4: Thermal stability test of hydrolysable tannins of TGII and PGG in methanol, ethanol, and water solutions. Table S5: Protective effects of different concentrations of ascorbic acid on TGII and PGG in a dry bath at 100 °C for 4 h.
