# Supplementary material for: Stability of Principal Hydrolysable Tannins from Trapa taiwanensis Hulls
Source: Molecules. 2019 Jan 21;24(2):365. doi: 10.3390/molecules24020365 (PMC6359050; doi:10.3390/molecules24020365)
Supplement: Supplementary file 1 [file molecules-24-00365-s001.pdf]

Supplementary Materials

Stability of Principal Hydrolysable Tannins from *Trapa taiwanensis* Hulls

Ching-Chiung Wang[1,2], Hsyeh-Fang Chen[3], Jin-Yi Wu[3], and Lih-Geeng Chen[3,*]

[1] School of Pharmacy, College of Pharmacy, Taipei Medical University, Taipei 11031, Taiwan; crystal@tmu.edu.tw
[2] Traditional Herbal Medicine Research Center, Taipei Medical University Hospital, Taipei 11031, Taiwan
[3] Department of Microbiology, Immunology and Biopharmaceuticals, College of Life Sciences, National Chiayi University, Chiayi 60004, Taiwan; snowfang@ccpc.com.tw, jywu@mail.ncyu.edu.tw, lgchen@mail.ncyu.edu.tw
* Correspondence: lgchen@mail.ncyu.edu.tw; Tel.: +886-5-2717798

Figure S1 The HPLC chromatograms of TGII (A) and PGG (B).

Table S1 The pH stability test of hydrolysable tannins of TGII and PGG. These hydrolysable tannins were treated with various pH values.

Table S2 Simulated gastric fluid and simulated intestinal fluid stability tests of the hydrolysable tannins of TGII and PGG.

Table S3 Photostability test of hydrolysable tannins of TGII and PGG.

Table S4 Thermal stability test of hydrolysable tannins of TGII and PGG in methanol, ethanol, and water solutions.

Table S5 Protective effects of different concentrations of ascorbic acid on TGII and PGG in a dry bath at 100 °C for 4 h.

(A)

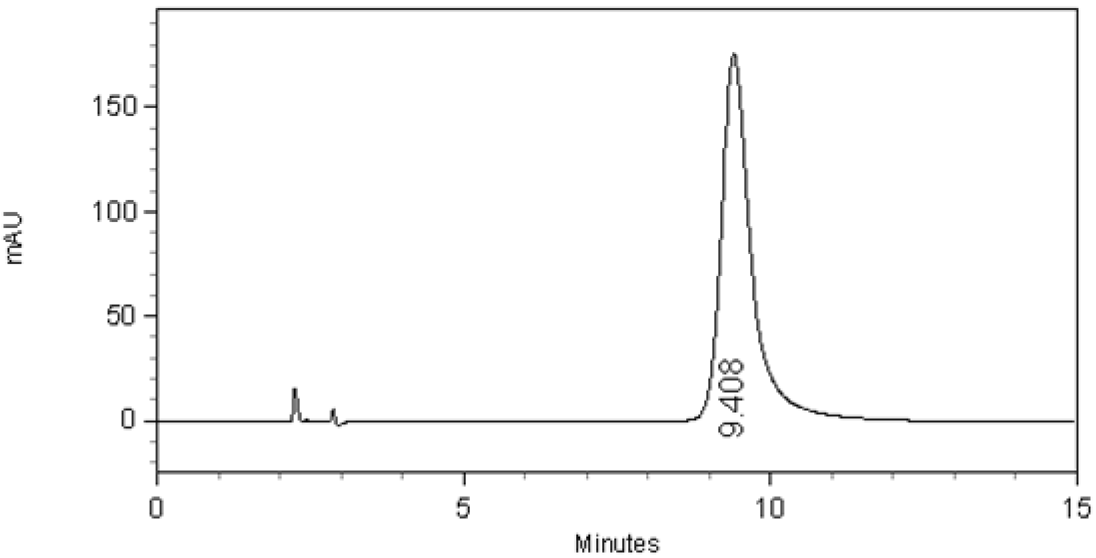

(B)

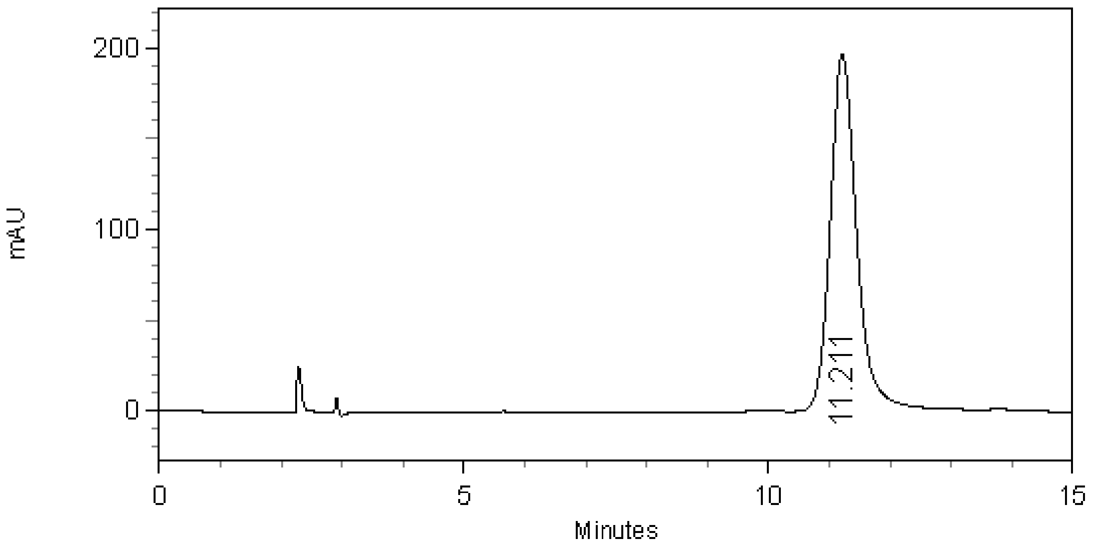

Figure S1. The HPLC chromatograms of TGII (A) and PGG (B). HPLC condition: mobile phase: 0.05 % TFA-CH_3CN (85 : 15); flow rate: 1.0 mL/min; column: LiChrospher® 100 RP-18e column (4 mm i.d. × 250 mm, 5 μm); column maintain at 40°C; detection: 280 nm.

Table S1. The pH stability test of hydrolysable tannins of TGII and PGG. These hydrolysable tannins were treated with various pH values.

pH	Time (h)					
	0	3	6	9	12	24
Sample: TGII (%)						
2	100.00 ± 1.44	104.15 ± 3.80	100.84 ± 0.81	101.94 ± 3.27	106.36 ± 0.78**	91.88 ± 0.38***
4	100.00 ± 1.46	85.23 ± 4.61**	87.43 ± 1.38***	86.86 ± 2.32***	80.56 ± 2.79***	60.20 ± 0.60***
6	100.00 ± 4.46	65.79 ± 0.20***	57.27 ± 1.81***	38.77 ± 1.11***	27.19 ± 0.37***	4.86 ± 0.05[a]
7	100.00 ± 1.18	65.10 ± 0.48***	40.61 ± 0.40***	25.39 ± 0.88***	14.04 ± 0.19***	1.71 ± 0.08[a]
8	100.00 ± 3.17	39.97 ± 1.05***	15.45 ± 0.61***	5.53 ± 0.07[a]	2.34 ± 0.19[a]	0.35 ± 0.06[a]
10	100.00 ± 3.49	0.00 ± 0.00[a]	0.00 ± 0.00[a]	0.00 ± 0.00[a]	0.00 ± 0.00[a]	0.00 ± 0.00[a]
Sample: PGG (%)						
2	100.00 ± 2.02	99.46 ± 1.06	105.92 ± 1.70**	105.71 ± 2.84*	108.41 ± 4.26*	101.86 ± 1.65
4	100.00 ± 4.40	87.97 ± 0.60**	92.98 ± 1.06*	91.10 ± 2.25*	83.52 ± 1.07**	75.32 ± 3.13***
6	100.00 ± 0.46	88.43 ± 1.56***	72.19 ± 1.04****	57.00 ± 1.66***	43.69 ± 1.12***	11.61 ± 0.15***
7	100.00 ± 0.75	71.81 ± 0.14***	61.62 ± 1.33***	43.30 ± 0.99***	32.18 ± 0.48***	3.22 ± 0.04[a]
8	100.00 ± 2.17	34.21 ± 0.10***	9.10 ± 0.23***	2.86 ± 0.19[a]	0.53 ± 0.05[a]	0.00 ± 0.00[a]
10	100.00 ± 2.09	22.04 ± 1.38***	0.00 ± 0.00[a]	0.00 ± 0.00[a]	0.00 ± 0.00[a]	0.00 ± 0.00[a]

Values are presented as the mean ± standard deviation. n=3.

* p<0.05, ** p<0.01, *** p<0.001 compared to the 0 h of sample.

[a] lower than limit of quantitation (LOQ).

Table S2. Simulated gastric fluid and simulated intestinal fluid stability tests of the hydrolysable tannins of TGII and PGG.

Simulated Gastric Fluid	Time (h)				
	0	1	2	3	4
TGII (%)	100.00 ± 1.87	103.01 ± 0.87*	93.93 ± 1.98**	102.22 ± 1.26	96.81 ± 0.55*
PGG (%)	100.00 ± 2.17	100.43 ± 1.36	105.97 ± 2.48*	104.90 ± 0.77*	103.35 ± 0.28*

Simulated Intestinal Fluid	Time (h)			
	0	3	6	9
TGII (%)	100.00 ± 2.43	84.47 ± 2.71***	56.79 ± 1.43***	31.40 ± 0.96***
PGG (%)	100.00 ± 1.67	74.74 ± 0.96***	37.55 ± 0.35***	12.46 ± 0.31***

Values are presented as the mean ± standard deviation. n=3.

* p<0.05, ** p<0.01, *** p<0.001 compared to the 0 h of sample.

Table S3. Photostability test of hydrolysable tannins of TGII and PGG.

Solvent	Time (h)				
	0	1	2	3	4
Sample: TGII (%)					
Methanol	100.00 ± 3.14	94.64 ± 2.25*	88.34 ± 2.18**	80.82 ± 2.17***	76.40 ± 1.31***
Ethanol	100.00 ± 2.77	87.13 ± 3.27**	79.80 ± 2.24***	71.90 ± 1.68***	60.98 ± 0.37***
Water	100.00 ± 0.55	99.09 ± 0.37*	97.67 ± 0.42**	96.46 ± 0.43***	95.05 ± 0.36***
Sample: PGG (%)					
Methanol	100.00 ± 0.83	101.21 ± 0.26	99.38 ± 2.16	91.28 ± 2.39**	83.00 ± 0.47***
Ethanol	100.00 ± 3.97	96.73 ± 2.67	89.30 ± 0.03**	80.24 ± 0.48***	72.74 ± 0.37***
Water	100.00 ± 0.65	100.50 ± 1.07	99.68 ± 0.38	99.01 ± 0.70	96.78 ± 0.30***

The sample solution was irradiated with an ultraviolent lamp of a photochemical reactor (8 W × 16 = 128 W) at 352 nm and a distance of about 3.2 cm for 4 h. n=3; values are presented as the mean ± standard deviation. * $p<0.05$, ** $p<0.01$, *** $p<0.001$ compared to the 0 h of sample.

Table S4. Thermal stability test of hydrolysable tannins of TGII and PGG in methanol, ethanol, and water solutions.

Temp. (°C)	Solvent	Time (h)				
		0	1	2	3	4
Sample: TGII (%)						
100	Methanol	100.00 ± 0.06	97.83 ± 0.89**	95.51 ± 0.51***	95.77 ± 0.58***	94.40 ± 1.83**
	Ethanol	100.00 ± 0.11	98.32 ± 0.25***	96.27 ± 0.97**	93.60 ± 0.44***	93.69 ± 0.32***
	Water	100.00 ± 0.85	70.94 ± 2.60***	47.72 ± 2.11***	37.03 ± 4.59***	40.19 ± 2.12***
90	Methanol	100.00 ± 0.06	98.44 ± 0.91*	98.12 ± 0.66**	95.81 ± 0.95***	97.06 ± 2.46
	Ethanol	100.00 ± 0.11	99.39 ± 3.13	96.43 ± 0.37***	95.39 ± 0.50***	95.64 ± 0.26***
	Water	100.00 ± 0.85	74.71 ± 1.37***	61.73 ± 2.80***	53.48 ± 1.85***	53.19 ± 2.81***
80	Methanol	100.00 ± 0.06	99.07 ± 0.29**	99.70 ± 0.14*	100.46 ± 3.84	96.78 ± 1.37**
	Ethanol	100.00 ± 0.11	99.70 ± 0.51	100.03 ± 0.34	96.43 ± 0.23***	96.23 ± 0.62***
	Water	100.00 ± 0.85	84.31 ± 1.50***	74.07 ± 0.75***	67.27 ± 0.28***	59.68 ± 1.36***
70	Methanol	100.00 ± 0.06	99.49 ± 0.68	98.77 ± 0.90*	98.02 ± 1.06*	97.72 ± 1.93
	Ethanol	100.00 ± 0.11	98.66 ± 0.33**	97.95 ± 0.32***	98.03 ± 0.75**	97.38 ± 0.40***
	Water	100.00 ± 0.85	85.36 ± 0.36***	79.97 ± 1.41***	76.49 ± 0.59***	70.94 ± 1.20***
Sample: PGG (%)						
100	Methanol	100.00 ± 0.96	99.21 ± 0.49	98.51 ± 1.03	100.58 ± 0.86	98.16 ± 2.33
	Ethanol	100.00 ± 1.84	101.78 ± 0.76	99.96 ± 1.15	99.34 ± 1.56	99.15 ± 0.84
	Water	100.00 ± 0.82	86.72 ± 4.13**	61.61 ± 3.01***	62.01 ± 3.32***	43.52 ± 1.72***
90	Methanol	100.00 ± 0.96	100.81 ± 1.45	98.09 ± 0.37*	98.82 ± 2.55	98.71 ± 2.41
	Ethanol	100.00 ± 1.84	102.23 ± 0.91	103.59 ± 0.31*	102.40 ± 0.50*	102.69 ± 0.22*
	Water	100.00 ± 0.82	92.34 ± 0.53***	84.82 ± 0.92***	76.26 ± 0.65***	68.46 ± 0.45***
80	Methanol	100.00 ± 0.56	102.74 ± 3.78	99.92 ± 1.39	99.16 ± 0.56	100.32 ± 3.19
	Ethanol	100.00 ± 0.40	99.70 ± 0.24	100.04 ± 0.63	99.70 ± 0.57	99.96 ± 1.61
	Water	100.00 ± 1.73	89.17 ± 2.74**	78.35 ± 2.58***	69.94 ± 0.65***	71.75 ± 4.13***
70	Methanol	100.00 ± 0.56	103.14 ± 2.48*	100.67 ± 0.97	102.77 ± 2.73	101.39 ± 1.36
	Ethanol	100.00 ± 0.40	99.22 ± 0.26*	100.03 ± 0.16	100.16 ± 0.46	99.59 ± 0.56
	Water	100.00 ± 1.73	91.94 ± 0.99**	85.80 ± 1.94***	83.47 ± 2.94***	83.48 ± 2.33***

n=3; values are presented as the mean ± standard deviation. * p<0.05, ** p<0.01, *** p<0.001 compared to the 0 h of sample.

Table S5 Protective effects of different concentrations of ascorbic acid on TGII and PGG in a dry bath at 100 °C for 4 h.

ascorbic acid (μg/mL)	TGII (%)	PGG (%)
Original, 0 hr	100.00 ± 0.40	100.00 ± 0.16
0	51.28 ± 0.16	54.29 ± 0.31
31.25	51.31 ± 0.58	55.62 ± 0.22
62.5	62.48 ± 0.62	59.01 ± 0.12
125	75.17 ± 0.28	61.55 ± 1.13
250	80.14 ± 0.36	65.09 ± 0.20
500	82.86 ± 0.11	64.51 ± 0.14
1000	85.55 ± 0.17	65.95 ± 0.20

n=3; values are presented as the mean ± standard deviation.
